# Non-invasive Metabolomic Profiling of Embryo Culture Medium Using Raman Spectroscopy With Deep Learning Model Predicts the Blastocyst Development Potential of Embryos

**DOI:** 10.3389/fphys.2021.777259

**Published:** 2021-11-19

**Authors:** Wei Zheng, Shuoping Zhang, Yifan Gu, Fei Gong, Lingyin Kong, Guangxiu Lu, Ge Lin, Bo Liang, Liang Hu

**Affiliations:** ^1^National Health Commission (NHC) Key Laboratory of Human Stem Cell and Reproductive Engineering, School of Basic Medical Science, Institute of Reproductive and Stem Cell Engineering, Central South University, Changsha, China; ^2^Clinical Research Center for Reproduction and Genetics in Hunan Province, Reproductive and Genetic Hospital of CITIC-Xangya, Changsha, China; ^3^Hunan International Scientific and Technological Cooperation Base of Development and Carcinogenesis, Changsha, China; ^4^Basecare Medical Device Co., Ltd., Suzhou, China; ^5^State Key Laboratory of Microbial Metabolism, Joint International Research Laboratory of Metabolic and Developmental Sciences, School of Life Sciences and Biotechnology, Shanghai Jiao Tong University, Shanghai, China

**Keywords:** embryo viability prediction, metabolomic profiling, multilayer perceptron, non-invasive assessment, Raman spectroscopy

## Abstract

**Purpose:** This study aimed to establish a non-invasive predicting model via Raman spectroscopy for evaluating the blastocyst development potential of day 3 high-quality cleavage stage embryos.

**Methods:** Raman spectroscopy was used to detect the metabolic spectrum of spent day 3 (D3) embryo culture medium, and a classification model based on deep learning was established to differentiate between embryos that could develop into blastocysts (blastula) and that could not (non-blastula). The full-spectrum data for 80 blastula and 48 non-blastula samples with known blastocyst development potential from 34 patients were collected for this study.

**Results:** The accuracy of the predicting method was 73.53% and the main different Raman shifts between blastula and non-blastula groups were 863.5, 959.5, 1,008, 1,104, 1,200, 1,360, 1,408, and 1,632 cm^–1^ from 80 blastula and 48 non-blastula samples by the linear discriminant method.

**Conclusion:** This study demonstrated that the developing potential of D3 cleavage stage embryos to the blastocyst stage could be predicted with spent D3 embryo culture medium using Raman spectroscopy with deep learning classification models, and the overall accuracy reached at 73.53%. In the Raman spectroscopy, ribose vibration specific to RNA were found, indicating that the difference between the blastula and non-blastula samples could be due to materials that have similar structure with RNA. This result could be used as a guide for biomarker development of embryo quality assessment in the future.

## Introduction

Infertility has become the third-most disease, after cancer and cardiovascular diseases. Infertility affects an estimated 8–12% of reproductive-age couples globally (Borght and Wyns, 2018). Since first human to have been born after *in vitro* fertilization (IVF) in July 25, 1978, assisted reproduction technology (ART) has gradually become the mainstream for treating infertility (Kamel, 2013). In conventional ART treatments, multiple–cleavage stage embryos were transferred together to increase the live birth rate, which, at the same time, increases the risks of preterm delivery, low birth weight, and dramatic cerebral palsy compared with a single embryo transfer (Scott et al., 2008). Advances in culture conditions and laboratory techniques have led to a shift from cleavage stage embryo transfer to blastocyst stage transfer in IVF to alleviate the serious side effects of multiple embryo transfer, improving both uterine and embryonic synchronicity and enabling self-selection of viable embryos for a better live birth rate (Blake et al., 2007). However, only about 50% of human embryos could develop to the blastocyst stage *in vitro* due to factors such as a high rate of development arrest (McCollin et al., 2020). Patients who do not have a sufficient number of high-quality embryos for extended culture to the blastocyst stage still persist to have their embryos transplanted on day 3 (D3) (Scott et al., 2008). In this scenario, a fast, reliable, and non-invasive evaluation method for differentiating viable and non-viable D3 embryos could largely improve the success rate of current IVF pregnancy.

The main current embryo assessment methodologies for IVF are morphological evaluation (Gardner and Balaban, 2016; Kaser et al., 2019). Embryo morphological observation, which is still the first-line approach for assessing embryo viability, observes the development pattern of embryos, including the timing and rate of cleavage, cell number, and fragmentation (Nadal-Desbarats et al., 2012; Kaser et al., 2019). Although it is quick, convenient, and inexpensive, morphological evaluation lacks an effectively unified evaluation standard (Sakkas and Gardner, 2013). It can be easily interfered by the subjective bias of different graders and complicated by different stages of embryonic development, rendering low predictive power and unreliable results (Milki et al., 2002; Guerif et al., 2007). Especially when all embryos have the same rating on day 3, it would be extremely confuse to select the viable embryo with great blastocyst development potential for transfer.

Evidence for the existence of metabolic differences between viable and non-viable embryos has been provided by several studies. Gardner et al. (2001) found that pyruvate and glucose uptake by day 4 was significantly higher by embryos that formed blastocysts than by embryos that failed to develop to the blastocyst stage. Zivi et al. (2014) suggested that decreased uptake of serine and possibly proline from the fertilization medium was associated with pregnancy and might be useful for embryo selection. High-resolution nuclear magnetic resonance (NMR) with a combination of probes indicated that *in vitro* cultured human embryos having a high developmental potential would modify their environment slightly compared with embryos that ceased to develop (Nadal-Desbarats et al., 2012). Thus, the metabolic profiling of spent embryo culture medium for the developmental potential of embryo blastocysts has been proposed as a rapid, non-invasive, sensitive, and, more importantly, clinically applicable approach (Liang et al., 2019).

Metabolomic profiling using Raman spectroscopy measures multiple small-molecule metabolites and their metabolite intermediates. It records the frequency shift in a biphotonic process, in which one molecule absorbs an incident photon and emits a Raman photon simultaneously. Because different molecules have different energy levels, which is reflected by different frequency shifts (wavenumber, cm^–1^), the individual bands in the Raman spectrum are the characteristic of specific molecular vibrations (Vargas-Obieta et al., 2016). In addition, comparing with other spectroscopy technologies, such as near-infrared spectroscopy, Raman spectroscopy is not sensitive to water interference and is advantageous for measuring analytes in cell cultures (Rowland-Jones et al., 2017). It can be used for the qualitative and quantitative detection of metabolites and is suitable for studying metabolic variation in microculture medium (Scott et al., 2008; Ishigaki et al., 2017). Metabolomic profiling with Raman spectroscopy has the advantage of simultaneously evaluating a large number of samples and providing a profile that may reflect embryo viability more clearly.

In this study, Raman spectra of the blastula and non-blastula samples were compared under the hypothesis that the metabolism of a healthy embryo might alter the surrounding environment differently from that of unhealthy embryos. In the Raman spectra obtained, only a small difference in intensity was observed between these two groups because an equal number of peaks and a close location of bands were observed in the spectral analysis. The principal component analysis with the spectral data showed no specific clustering pattern for the first three principal components, indicating no detectable difference between the blastula and non-blastula samples. Further analysis with the deep learning model could find subtle differences between the two groups of samples by scrutinizing more than 1,000 features of the Raman spectrum. This deep learning model constructed with Raman spectral data achieved an overall accuracy of 73.53% for predicting the blastocyst development potential of embryos, indicating the strength of combing Raman spectroscopy with deep learning algorithms in assessing the viability of embryos *in vitro*. Moreover, the linear discriminant analysis with the spectra data of all samples showed that the Raman shifts were mainly associated with ribose bond vibration that specific to RNA, thus guiding biomarker development for embryo quality assessment to the direction of materials with similar structures with RNA.

## Materials and Methods

### Patients and Treatment

In this retrospective study, 34 patients adopted preimplantation genetic testing for aneuploidies (PGT-A) were recruited without any selectivity from Jan, 2020 to Feb, 2020 at the Reproductive and Genetic Hospital of CITIC-XIANGYA. Their 80 embryos that developed to the blastocyst stage and 48 embryos that did not develop to the blastocyst stage were included.

### Embryo Culture and Sample Collection

As for PGT-A, the insemination method of intracytoplasmic sperm injection (ICSI) was selected to avoid the interference of cumulus cells during the biopsy process. ICSI mainly can be summarized into two steps, cumulus cell removal to evaluate oocyte nuclear maturity and single live sperm injection into the center of oocyte. The embryos were cultured singly with 25 μL cleavage medium (SICM, G19018, Cook Medical, Bloomington, United States) culture medium in each well covered with OVOIL (10029, Vitrolife, Göteborg, Sweden). On day 3 after fertilization, the embryos that had developed into six to eight cells were transferred to blastocyst culture medium (SIBM, G46296, Cook Medical, Bloomington, United States). After removing embryos, 15 μL of the SICM culture medium was collected in an RNase-DNase-free PCR tube as a D3 culture medium sample and stored in a refrigerator at –80°C. As indicated in our previous paper, there was no observable difference in Raman spectra between the fresh samples and the samples stored at –80°C (Liang et al., 2019). And all samples were stored for an equivalent period so that the time of storage could not be a confounding factor to the result.

### Raman Spectroscopy

Raman spectra were detected using Witec alpha300 R confocal Raman spectroscopy (WITec Wissenschaftliche Instrumente und Technologie GmbH, Ulm, Germany) with 532-nm excitation wavelength, 1,200 g/mm grating, and 5 mW power. D3 spent embryo culture medium samples frozen at –80°C were thawed at room temperature (25°C) for 30 min. A specifically designed sampler took 2 μL from each sample and placed the droplets onto a quartz glass slide coated with a thin layer (100 nm) of aluminum film substrate carefully. After natural drying, there was a stacking pattern formed, which was the so-called “coffee-ring” (Luo et al., 2016). To avoid variation from natural drying of the droplets, five points were randomly selected from the coffee-ring. Raman signals were obtained from those points selected by confocal Raman microscopy, yielding 400 blastula spectra and 240 non-blastula spectra. Each point was accumulated eight times and integrated with a charge-coupled device camera with a duration of 8 s. The Raman spectra were recorded in a spectral range of 60–2,000 cm^–1^ with a spectral resolution of 1 cm^–1^. It was approximately 10 min per sample detection.

### Data Analysis

All spectra data were preprocessed using WITec Suite FIVE version 5.1 (WITec Wissenschaftliche Instrumente und Technologie GmbH, Ulm, Germany) and then saved as raw data after removing the background fluorescence signal. These raw data were then transformed by a B-spline interpolation method into a Raman displacement interval of 1 cm^–1^. Vector normalization was performed to normalize the Raman spectrum data in the 200–2,000 cm^–1^ range, and the biological fingerprinting range of Raman shift (600–1,800 cm^–1^) was extracted for subsequent analysis. Signals from the SICM culture medium were treated as background control spectrum and was subtracted from each sample spectrum with the same methods as described in our previous study (Liang et al., 2019). Unsupervised clustering method, Principal Component Analysis (PCA), was used to detect whether data could be clustered into different groups. As a popular dimensionality reduction algorithm, PCA would identify the hyperplane that lies closet to the data, and then project data onto the hyperplane, transforming data into different axes (Jolliffe and Cadima, 2016).

### Multilayer Perceptron

The simplest deep network, multilayer perceptron (MLP), was implemented in this study. It consisted of multiple layers of neurons, each fully connected to neurons in the layers from which they received input and to neurons in the layers that they influenced. The schema of the network comprised one input layer for Raman spectral input, one output layer for the predicted result, and two dense layers between the input and output layers. Each dense layer contains 64 neurons. In the training stage, the input were the full spectrum data (600–1,800 cm^–1^) of Raman spectroscopy from all samples in the training dataset; in the validation stage, the input were the full spectrum data of Raman spectroscopy from all samples in the validation dataset. The activation function, loss function, and optimizer for MLP were the rectified linear unit function, cross-entropy, and the Adam optimizer, respectively. The training cohort was randomly chosen from both blastula and non-blastula samples, with 30 D3 spent culture medium samples from both groups. As described in the “Materials and Methods” section, each sample would generate five spectral results. A total of 300 spectra, comprising 150 blastula and 150 non-blastula spectra, were used in the MLP model training. The remaining samples in each group, 50 blastula and 18 non-blastula samples, were used as the testing cohort. In total, 340 Raman spectra (250 blastula and 90 non-blastula spectra) were predicted and evaluated with the MLP model.

In the MLP model, the 10 × cross-validation method was used for verification model training. During the model training process, the loss status of the verification set was monitored. When it reached a stable level, the training was completed. Then, the verification set was used to build the model. The testing cohort examined the model performance verification. The overfitting of the deep learning model was thus reduced by this general method, and the universal ability of the model was improved at the same time. The performance of the model was evaluated with sensitivity, specificity, and accuracy calculated from the predicting results and the known status of each testing sample. All the codes were built with Python 3.5.2.

### Characteristic Raman Shift Screening

Raman spectra are characterized by a shift in wave numbers (inverse of wavelength in cm^–1^) (Talari et al., 2014). The linear discriminant analysis (LDA) was used to reduce the dimensions of the original Raman spectral data. The results of dimensionality reduction were analyzed by one-dimensional cluster analysis and characteristic Raman shift screening. The Raman characteristic shifts with an absolute correlation coefficient greater than 3 were selected and annotated according to the published literature. The data processing was performed in R 3.6.

## Results

### Clinical Characteristics

**FIGURE 1:**
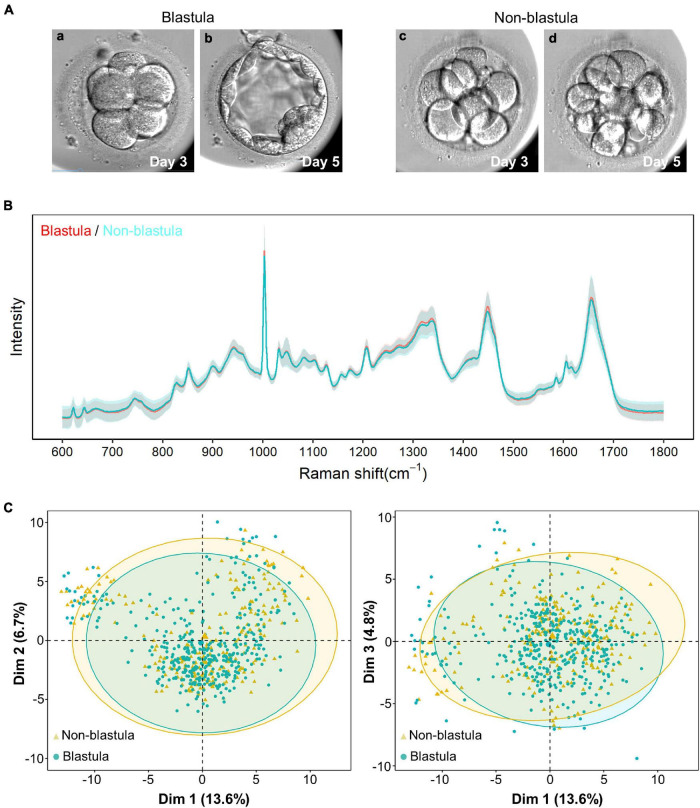
Raman spectrum and unsupervised clustering with principal component analysis (PCA). **(A)** The pattern diagram of blastula and non-blastula group. The cleavage stage embryos are similar at day 3 **(a,c)**, the cleavage stage embryos developed to blastocysts **(b)** in blastula groups, yet arrested at cleavage stage **(d)** in non-blastula group. **(B)** The intensity from both the blastula and non-blastula group are plotted vs. the Raman shift. Solid line represents the average of the Raman spectrum data, and the shading depicts the standard deviation of the Raman spectrum. The average Raman spectrum intensity of the blastula group is depicted by a red line, and the standard deviation of this group is depicted by light red. The color used for the non-blastula group is blue for the average Raman spectrum intensity line, and light blue for the standard deviation. Most part of the two lines overlap with each other, where only blue line is visible. The parts that both lines are shown indicate places where the groups are different. The shading for both groups overlap with each other most of the time, indicating that both groups had similar variations. **(C)** Unsupervised clustering using PCA showed no specific pattern for the first three principal components. Dim1, Dim2, and Dim3 are the first, second and third principal components, respectively. The numbers in parentheses represent the contribution ratio of the corresponding principal components. The ellipses of the clusters are drawn according to the 95% confidence interval of the data. The blastula and non-blastula group are highly consistent with the first three dimension, as shown by the first three principal components.

To avoid the influence of cumulus cells metabolism, all the recruited 34 patients adopted preimplantation genetic testing for aneuploidies prior to embryos transfer, so we could observe the blastocyst formation of these embryos. High quality cleavage embryos, which contained seven or eight blastomeres on D3 were included in this study. 80 of 128 embryos mentioned above formed blastocysts during subsequent blastocyst culture, which defined as group “blastula,” the others were as group “non-blastula.” During the 34 patients, 10 of which were totally categorized to group “non-blastula,” and seven of which to group “blastula” (Figure 1A and Supplementary Table 1), we further analyzed the essential characteristics of patients to above two tendencies of embryonic development, and found only the young maternal age and more antral follicle count contributed to higher embryonic development potential (Supplementary Table 2).

### Detection of D3 Embryo Spent Culture Based on High-Resolution Raman Spectroscopy

Full Raman spectral data were obtained from 128 D3 spent embryo culture media by high-precision confocal Raman spectroscopy. The biological fingerprinting area of 600–1,800 cm^–1^ in Raman shift was extracted from each spectrum for subsequent analysis. As shown in Figure 1B, the blastula sample depicted by a red line had only a slight visual difference compared with the non-blastula sample depicted by a blue line. The number of characteristic peaks was equal, 29 peaks in each group; the difference resided mainly in the intensity. To detect whether data from the blastula and non-blastula group could be categorized into two different groups, PCA was applied. However, no obvious clustering phenomenon was found by this unsupervised clustering algorithm (Figure 1C). The reason for such a small difference could be that the main metabolite components in the D3 culture medium were derived only from eight cells so that changes in the medium were relatively small.

### Deep Learning Model Based on Multilayer Perceptron to Evaluate Embryonic Potential

Machine learning was used in the analysis to differentiate between the blastula and non-blastula samples with a higher resolution. In the training dataset, the spectra data of 30 blastula samples (5 spectra data for each sample and 150 spectra data for the blastula samples) and 30 non-blastula samples (150 spectra data for the non-blastula samples) were randomly selected from 80 blastula and 48 non-blastula samples (640 spectral data in total). Detailed information about each sample, including whether the embryo developed to the blastocyst stage, which group had the sample been assigned to, and the predicting result for the sample in the testing cohort. The overall performance of the prediction is shown in Table 1. Of the 18 non-blastula samples, 14 were successfully predicted to be non-blastula, resulting in a sensitivity of 77.78%. Of the 50 blastula embryos, 36 were successfully predicted to be blastula, resulting in a specificity of 72.00%. The overall accuracy of the prediction was 73.53%, indicating that most of the samples were correctly classified.

**TABLE 1:** Performance of the deep learning model.

		Predicted	
		Non-blastula	Blastula
Real	Non-blastula	14	4
	Blastula	14	36

Sensitivity		77.78%	
Specificity		72.00%	
Accuracy		73.53%	

### Differential Raman Shift Screening

**FIGURE 2:**
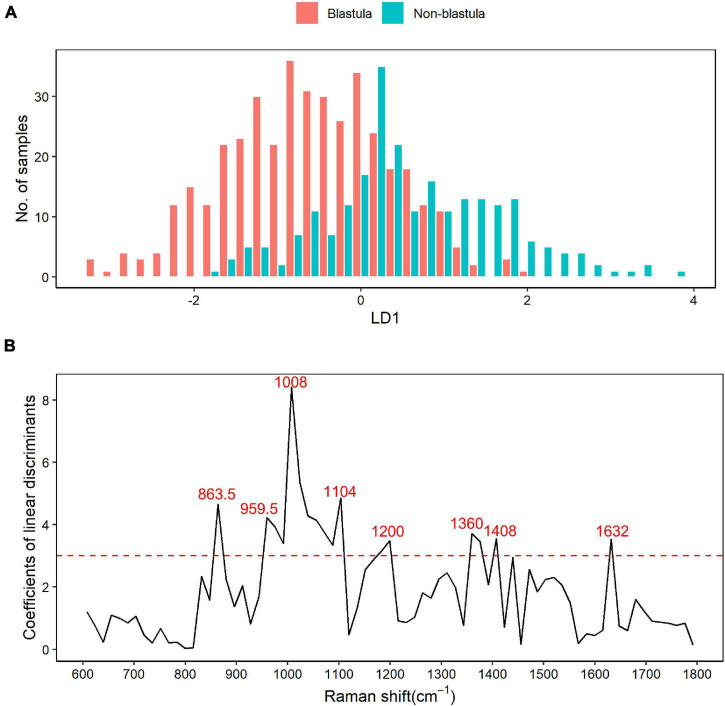
Raman shift based on the linear discriminant analysis (LDA). **(A)** Result of linear discriminant analysis. LDA for two categories, blastula or non-blastula, results in one linear discriminant factor (LD1). The number of samples across each LD1 in both groups are plotted in the figure, with the blastula group depicted by red bars and the non-blastula group depicted by blue bars. It shows that the blastula and non-blastula samples had a lot of overlaps within the range from –2 to 2 of LD1, while no overlap is observed outside that range. **(B)** Raman shifts screened by LDA. The coefficients of linear discriminants are plotted against Raman shifts. Raman shifts with a correlation coefficient greater than 3 were selected as bands with a large contribution to the difference between groups of samples based on rule of thumb. With this criterion, eight different Raman shifts are chosen as the main different components between the two groups.

LDA differential Raman shift screening was carried out within the 600–1,800 cm^–1^ biological fingerprinting region, and the results were annotated with the published literature. The histogram from LD1 showed that the blastula and non-blastula samples had a lot of overlap, as shown in Figure 2A, which made the separation of these two groups of samples difficult. As depicted in Figure 2B, in the LDA analysis, Raman shifts with a correlation coefficient greater than 3 were selected as bands with a large contribution to the difference between groups of samples. In total, eight wave ranges with significant differences were screened out with this criterion. The main different Raman shifts were 863.5, 959.5, 1,008, 1,104, 1,200, 1,360, 1,408, and 1,632 cm^–1^. The highest peak in Figure 2B was the Raman shift at 1,008 cm^–1^. The annotated results of differential characteristic wave numbers are shown in Table 2.

**TABLE 2:** Main bands observed and corresponding assignment of biomolecules.

Raman shift (cm^–1^)	Chemical bond	Substrate
863.5	Ribose vibration	RNA
959.5	Symmetric stretching vibration of v_1–_PO^3–^_4_ Calcium-phosphate stretching band	Phosphate of HA cholesterol
1,008	Aromatic ring breathing, v(CO), v(CC), OCH	Any molecule with a benzene ring
1,104	C-C-H bending, carbohydrate peak for solutions	Phenylalanine proteins
1,200	Aromatic C-O and C-N	Nucleic acids and phosphates
1,360	–	Tryptophan
1,408	COO^–^	IgG
1,632	C = O	Amide I

## Discussion

In this study, we developed a non-invasive cleavage embryo selection method based on Raman spectroscopy. Although the current accuracy reached at 73.53%, it may combine with traditional morphological evaluation to select a higher blastocyst development potential blastocyst development potential embryos for transfer.

This was different from several other studies that also used the Raman spectrum to assess embryos’ viability in IVF. We previously detected a marked difference between aneuploid and euploid embryos in the mean-centered spectral analysis (Liang et al., 2019). In the Raman spectra analysis to differentiate between embryos that were implanted and led to delivery and those that were not implanted, Seli et al. (2007) also found between-group differences in their mean-centered intensity analysis with 69 D3 spent embryo culture media from 30 patients. The same group also found spectral differences between the spent media of those that were implanted compared with those that failed to be implanted (Scott et al., 2008). The difference between this study herein and other studies could probably be attributed to the difference in the grouping of samples. In this study, whether the embryo could develop to the blastocyst stage was used as the grouping criterion. Another factor that might contribute to the difference would be the timing of culture media. Since early embryonic development has gone through two stages, the cleavage stage (day 1-day 3 post insemination) and the blastocyst formation stage (day 3-day 6 post insemination). For example, day 5 and day 6 spent embryo culture media were detected to predict the euploidy in blastocysts in our previous study (Liang et al., 2019), while only day 3 spent embryo culture media were used in the present study to predict the blastocysts formation. Still another factor for the difference could be the sample size; both studies conducted by Seli’s group had a small sample size (fewer than 50 samples), while 128 samples, each with five spectral data, were used for analysis in this study. No significant differences in spectral intensity analysis and mean-centered intensity analysis (data not shown) with data were found between the blastula and non-blastula samples because they had the same Raman shifts within the 600–1,800 biological fingerprint region with only a slight difference in intensity.

Raman spectra contained more than 1,000 features that could be used for analysis (Liang et al., 2019); with deep learning networks, all the features could be used for the differentiation between the blastula and non-blastula samples. This method combining deep learning network with Raman spectra to predict the embryo development to the blastocyst stage in the present study achieved an overall accuracy close to 74%, with a sensitivity of 77.78% and a specificity of 72%. Based on Raman metabolomic profiling of D3 spent culture media samples, higher embryo viability scores were found in embryos with proven reproductive potential in the study by Scott et al. (2008). The overall accuracy for predicting delivery or a failed implantation in the study by Scott et al. (2008) was 80.5%, the sensitivity was 85.7%, and the specificity was 80%. The reason for the discrepancy in performance between the study by Scott et al. (2008) and our study was related to sample size and differential grouping as discussed earlier. The study by Scott et al. (2008) included only 41 spectral media samples from 19 patients, and the 2 groups comprised embryos that were implanted and that failed to be implanted. The 128 samples with 5 Raman spectra each in this study outnumbered those in the study by Scott et al. (2008) Hence, a larger sample size needs to be used in the training stage to improve the accuracy of the predicting algorithm in this MLP model in our further research.

Additionally, the correlations between Raman shifts and nucleic acids were found, indicating that nucleic acids might be an important metabolic component in blastocyst formation. In this study, eight wave ranges with significant differences were found between the blastula and non-blastula samples, of which 863.5 cm^–1^ was related to RNA ribose chemical bond vibration (Talari et al., 2014; Potara et al., 2020), and 959.5 and 1,200 cm^–1^ were related to the vibration of the ribonucleic acid phosphate group (Schulz et al., 2003; Talari et al., 2014; Dooley et al., 2020; Potara et al., 2020). A significantly higher intensity peak was observed at 1,008 cm^–1^, indicating a resonance peak of the benzene ring (Talari et al., 2014; Potara et al., 2020), suggesting that substances with a benzene ring structure were different between the blastula and non-blastula samples. However, all substances with a benzene ring would produce a significant Raman peak at around 1,008 cm^–1^; this peak did not provide direct evidence regarding phenylalanine. Other substances containing the benzene ring structure, such as proteins and peptides, could also produce such a peak, making the biological interpretation of this peak difficult. The peaks at 1,360, 1,408, and 1,632 cm^–1^ were related to tryptophan, COO-, and Amide I, respectively (Talari et al., 2014; Kuhar et al., 2018; Signorelli et al., 2019; Potara et al., 2020). Apart from the peaks related to benzene ring structures, the remaining main peaks, 863.5, 959.5, and 1,200 cm^–1^ were mostly related to ribonucleic acid molecules, indicating that RNA nucleic acid might play an important role in differentiating embryos that could develop to the blastocyst stage and embryos that could not. This result was reasonable because miRNAs were expressed throughout embryonic cellular divisions and embryonic genome activation, and small RNAs could be secreted into embryo culture media (Kropp et al., 2014).

This implied that the role of materials that have similar structure with RNA in differentiating embryos with different blastocyst development potential was consistent with that in several other studies. We previously reported that the overall pattern of PCA loading at one dimension was close to the Raman spectrum of small RNA in PCA analysis of Raman spectrum of spent embryo culture media samples with known PGT-A results (Liang et al., 2019). Moreover, (Kropp et al. (2014) showed that miRNAs were secreted from pre-implantation embryos into culture media and that miRNA expression might correlate with the developmental competence of the embryo. In their study, differential miRNA gene expression, including miR-25, miR-302c, miR-196a2, and miR-181a, was observed between embryos that developed to the blastocyst stage and those that failed to develop from the morula to the blastocyst stage in bovine embryos; these miRNAs were also expressed in the culture media of both bovine and human models (Kropp et al., 2014). Given these results and the main peaks that were related to RNA nucleic acid in this study, it is natural to reason that the expression of miRNAs in *in vitro* culture media could allow for the development of biological markers for selecting better-quality embryos.

## Conclusion

In conclusion, these findings resonated with the previous finding that embryos with greater reproductive potential impacted their environment differently from those with lesser potential. These findings also verified the applicability of Raman spectroscopy combined with the deep learning model in biological detection and provided a target research direction of RNA nucleic acid–related molecules for a follow-up biomarker study.

## Data Availability Statement

The raw data supporting the conclusions of this article will be made available by the authors, without undue reservation.

## Ethics Statement

The studies involving human participants were reviewed and approved by the Reproductive and Genetic Hospital of CITIC-XIANGYA (Research license LL-SC-2018-037). The patients/participants provided their written informed consent to participate in this study.

## Author Contributions

LH, GLi, and WZ designed the research. BL and LK analyzed the data. BL and WZ wrote the manuscript draft. WZ edited the article. SZ and YG contributed to the sample collection. FG and GLu contributed to important intellectual content. All authors read and approved the final manuscript.

## Conflict of Interest

BL was employed by company Basecare Medical Device Co., Ltd. The remaining authors declare that the research was conducted in the absence of any commercial or financial relationships that could be construed as a potential conflict of interest.

## Publisher’s Note

All claims expressed in this article are solely those of the authors and do not necessarily represent those of their affiliated organizations, or those of the publisher, the editors and the reviewers. Any product that may be evaluated in this article, or claim that may be made by its manufacturer, is not guaranteed or endorsed by the publisher.

## References

[B1] Blake D. Farquhar C. Johnson N. Proctor M. (2007). Cleavage stage versus blastocyst stage embryo transfer in assisted reproductive technology. *Cochrane Database Systematic Rev.* 30:CD002118. 10.1002/14651858.CD002118.pub3 17943767

[B2] Borght M. V. Wyns C. (2018). Fertility and infertility: definition and epidemiology. *Clin. Biochem.* 62 2–10. 10.1016/j.clinbiochem.2018.03.012 29555319

[B3] Dooley M. Mclaren J. Rose F. Notingher I. (2020). Investigating the feasibility of spatially offset Raman spectroscopy for in-vivo monitoring of bone healing in rat calvarial defect models. *J. Biophoton.* 13:e202000190. 10.1002/jbio.202000190 32658374

[B4] Gardner D. K. Lane M. Stevens J. Schoolcraft W. B. (2001). Noninvasive assessment of human embryo nutrient consumption as a measure of developmental potential. *Fertil. Steril.* 76 1175–1180. 10.1016/s0015-0282(01)02888-6 11730746

[B5] Gardner K. D. Balaban B. (2016). Assessment of human embryo development using morphological criteria in an era of timelapse, algorithms and ‘OMICS’: is looking good still important? *Mol. Hum. Reprod.* 22 704–718. 10.1093/molehr/gaw057 27578774

[B6] Guerif F. Gouge A. L. Giraudeau B. Poindron J. Bidault R. Gasnier O. (2007). Limited value of morphological assessment at days 1 and 2 to predict blastocyst development potential: a prospective study based on 4042 embryos. *Hum. Reprod.* 22 1973–1981. 10.1093/humrep/dem100 17496054

[B7] Ishigaki M. Hashimoto K. Sato H. Ozaki Y. (2017). Non-destructive monitoring of mouse embryo development and its qualitative evaluation at the molecular level using Raman spectroscopy. *Sci. Rep.* 7:43942. 10.1038/srep43942 28272511 PMC5341076

[B8] Jolliffe I. T. Cadima J. (2016). Principal component analysis: a review and recent developments. *Philos. Trans. R. Soc. A: Mathematical Phys. Eng. Sci.* 374:20150202. 10.1098/rsta.2015.0202 PMC4792409 26953178

[B9] Kamel R. M. (2013). Assisted reproductive technology after the birth of louise brown. *J. Reprod. Infertil.* 14 96–109. 24163793 PMC3799275

[B10] Kaser D. J. Ginsburg E. S. Carrell D. T. Racowsky C. (2019). “Chapter 31 - Assisted reproduction,” in *Yen and Jaffe’s Reproductive Endocrinology (Eighth Edition)*, eds Strauss J. F. Barbieri R. L. (Philadelphia, PA: Content Repository Only!), 779–822.e716.

[B11] Kropp J. Salih S. M. Khatib H. (2014). Expression of microRNAs in bovine and human pre-implantation embryo culture media. *Front. Genet.* 5:91. 10.3389/fgene.2014.00091 24795753 PMC4006060

[B12] Kuhar N. Sil S. Verma T. Umapathy S. (2018). Challenges in application of Raman spectroscopy to biology and materials. *RSC Adv.* 8 25888–25908. 10.1039/c8ra04491k PMC9083091 35541973

[B13] Liang B. Gao Y. Xu J. Song Y. Xuan L. Shi T. (2019). Raman profiling of embryo culture medium to identify aneuploid and euploid embryos. *Fertil. Steril.* 111:e751. 10.1016/j.fertnstert.2018.11.036 30683589

[B14] Luo Q. Li X. Gu Y. Tang Y. Tan Z. Chen D. (2016). “A method based on coffee-ring deposition confocal Raman spectroscopy of analysis of melamine in milk,” in *Proceedings of the SPIE 10024, Optics in Health Care and Biomedical Optics VII* (Bellingham, DC).

[B15] McCollin A. Swann R. L. Summers M. C. Handyside A. H. Ottolini C. S. (2020). Abnormal cleavage and developmental arrest of human preimplantation embryos in vitro. *Eur. J. Med. Genet.* 63:103651. 10.1016/j.ejmg.2019.04.008 30995534

[B16] Milki A. A. Hinckley M. D. Gebhardt J. Dasig D. Westphal L. M. Behr B. (2002). Accuracy of day 3 criteria for selecting the best embryos. *Fertil. Steril* 77 1191–1195. 10.1016/s0015-0282(02)03104-7 12057727

[B17] Nadal-Desbarats L. Veau S. Blasco H. Emond P. Royere D. Andres C. R. (2012). Is NMR metabolic profiling of spent embryo culture media useful to assist in vitro human embryo selection? *Magnetic Resonance Mater. Phys. Biol. Med.* 26 193–202. 10.1007/s10334-012-0331-x 22878530

[B18] Potara M. Campu A. Maniu D. Focsan M. Botiz I. Astilean S. (2020). “Advanced nanostructures for microbial contaminants detection by means of spectroscopic methods,” in *Advanced Nanostructures for Environmental Health*, eds Baia L. Pap Z. Baia M. Hernadi K. (Amsterdam: Elsevier), 347–384.

[B19] Rowland-Jones R. C. Van Den Berg F. Racher A. J. Martin E. B. Jaques C. (2017). Comparison of spectroscopy technologies for improved monitoring of cell culture processes in miniature bioreactors. *Biotechnol. Prog.* 33 337–346. 10.1002/btpr.2459 28271638 PMC5413828

[B20] Sakkas D. Gardner D. K. (2013). “Limitations and benefits of morphologic embryo assessment strategies: how far can morphological assessment go in the identification of viable embryos?” in *Human Gametes and Preimplantation Embryos*, eds Gardner D. Sakkas D. Seli E. Wells D. (New York, NY: Springer).

[B21] Schulz H. Schrader B. Quilitzsch R. Pfeffer S. Kruger H. (2003). Rapid classification of basil chemotypes by various vibrational spectroscopy methods. *J. Agric. Food Chem.* 51 2475–2481. 10.1021/jf021139r 12696923

[B22] Scott R. Seli E. Miller K. Sakkas D. Scott K. Burns D. H. (2008). Noninvasive metabolomic profiling of human embryo culture media using Raman spectroscopy predicts embryonic reproductive potential: a prospective blinded pilot study. *Fertil. Steril.* 90 77–83. 10.1016/j.fertnstert.2007.11.058 18281045

[B23] Seli E. Sakkas D. Scott R. Kwok S. C. Rosendahl S. M. Burns D. H. (2007). Noninvasive metabolomic profiling of embryo culture media using Raman and near-infrared spectroscopy correlates with reproductive potential of embryos in women undergoing in vitro fertilization. *Fertil. Steril.* 88 1350–1357. 10.1016/j.fertnstert.2007.07.1390 17923129

[B24] Signorelli S. Cannistraro S. Bizzarri A. R. (2019). Raman evidence of p53-DBD disorder decrease upon interaction with the anticancer protein azurin. *Int. J. Mol. Sci.* 20:3078. 10.3390/ijms20123078 31238511 PMC6627904

[B25] Talari A. C. S. Movasaghi Z. Rehman S. Rehman I. U. (2014). Raman spectroscopy of biological tissues. *Appl. Spectroscopy Rev.* 50 46–111.

[B26] Vargas-Obieta E. Martinez-Espinosa J. C. Martinez-Zerega B. E. Jave-Suarez L. F. Aguilar-Lemarroy A. Gonzalez-Solis J. L. (2016). Breast cancer detection based on serum sample surface enhanced Raman spectroscopy. *Lasers Med. Sci.* 31 1317–1324. 10.1007/s10103-016-1976-x 27289243

[B27] Zivi E. Barash D. Aizenman E. Gibson D. Shufaro Y. (2014). Zygote serine decreased uptake from the fertilization medium is associated with implantation and pregnancy. *J. Assist. Reprod. Genet.* 31 889–897. 10.1007/s10815-014-0231-2 24789167 PMC4096884

