# Supplementary material for: Non-invasive Metabolomic Profiling of Embryo Culture Medium Using Raman Spectroscopy With Deep Learning Model Predicts the Blastocyst Development Potential of Embryos
Source: Front Physiol. 2021 Nov 19;12:777259. doi: 10.3389/fphys.2021.777259 (PMC8640355; doi:10.3389/fphys.2021.777259)
Supplement: Supplementary file 1 [file Table_1.DOCX]

Supplementary Table 1. Sample information and related role in model construction

| **Embryo_ID** | **Group** | **Training_Set** | **Testing_Set** | **Predicted_Result** |
| --- | --- | --- | --- | --- |
| N1-1-G | Blastula |  | Testing | Nonblastula |
| N1-6-G | Blastula |  | Testing | Blastula |
| N1-3-G | Blastula |  | Testing | Blastula |
| N2-7-B | Nonblastula |  | Testing | Blastula |
| N2-6-G | Blastula |  | Testing | Blastula |
| N2-10-G | Blastula |  | Testing | Blastula |
| N3-5-G | Blastula |  | Testing | Blastula |
| N3-3-G | Blastula |  | Testing | Blastula |
| N3-2-G | Blastula |  | Testing | Blastula |
| N3-15-G | Blastula |  | Testing | Blastula |
| N3-14-G | Blastula |  | Testing | Blastula |
| N3-9-G | Blastula | Training |  |  |
| N3-16-B | Nonblastula | Training |  |  |
| N4-3-G | Blastula | Training |  |  |
| N4-2-G | Blastula | Training |  |  |
| N4-11-G | Blastula | Training |  |  |
| N5-7-B | Nonblastula | Training |  |  |
| N5-6-B | Nonblastula | Training |  |  |
| N5-5-B | Nonblastula | Training |  |  |
| N6-2-B | Nonblastula | Training |  |  |
| N6-10-G | Blastula | Training |  |  |
| N7-5-G | Blastula | Training |  |  |
| N7-3-G | Blastula | Training |  |  |
| N7-2-G | Blastula | Training |  |  |
| N7-13-B | Nonblastula | Training |  |  |
| N10-9-B | Nonblastula |  | Testing | Nonblastula |
| N10-6-B | Nonblastula |  | Testing | Nonblastula |
| N10-18-B | Nonblastula |  | Testing | Nonblastula |
| N10-15-B | Nonblastula |  | Testing | Nonblastula |
| N10-21-G | Blastula |  | Testing | Blastula |
| N10-19-G | Blastula |  | Testing | Blastula |
| N10-16-G | Blastula |  | Testing | Blastula |
| N10-14-B | Nonblastula |  | Testing | Blastula |
| N15-5-B | Nonblastula |  | Testing | Nonblastula |
| N15-19-B | Nonblastula |  | Testing | Nonblastula |
| N15-8-G | Blastula |  | Testing | Blastula |
| N15-2-G | Blastula |  | Testing | Blastula |
| N15-13-G | Blastula |  | Testing | Blastula |
| N15-12-G | Blastula |  | Testing | Blastula |
| N15-10-B | Nonblastula |  | Testing | Blastula |
| N16-6-G | Blastula |  | Testing | Nonblastula |
| N16-5-B | Nonblastula |  | Testing | Nonblastula |
| N16-4-B | Nonblastula |  | Testing | Nonblastula |
| N16-3-G | Blastula |  | Testing | Blastula |
| N16-1-G | Blastula |  | Testing | Blastula |
| N17-11-G | Blastula |  | Testing | Nonblastula |
| N17-5-B | Nonblastula |  | Testing | Nonblastula |
| N17-15-B | Nonblastula |  | Testing | Nonblastula |
| N17-14-B | Nonblastula |  | Testing | Nonblastula |
| N17-1-B | Nonblastula |  | Testing | Nonblastula |
| N17-3-B | Nonblastula |  | Testing | Blastula |
| N17-16-G | Blastula |  | Testing | Blastula |
| N18-4-B | Nonblastula |  | Testing | Nonblastula |
| N18-2-G | Blastula |  | Testing | Nonblastula |
| N18-1-G | Blastula |  | Testing | Nonblastula |
| N19-6-G | Blastula |  | Testing | Nonblastula |
| N19-2-G | Blastula |  | Testing | Nonblastula |
| N19-12-G | Blastula |  | Testing | Nonblastula |
| N21-3-B | Nonblastula |  | Testing | Nonblastula |
| N21-2-B | Nonblastula | Training |  |  |
| N22-8-G | Blastula |  | Testing | Nonblastula |
| N22-3-B | Nonblastula | Training |  |  |
| N23-5-G | Blastula |  | Testing | Nonblastula |
| N23-6-B | Nonblastula | Training |  |  |
| N24-7-G | Blastula |  | Testing | Nonblastula |
| N24-4-G | Blastula |  | Testing | Nonblastula |
| N24-1-G | Blastula |  | Testing | Blastula |
| N25-9-G | Blastula |  | Testing | Nonblastula |
| N25-5-G | Blastula |  | Testing | Blastula |
| N25-2-G | Blastula |  | Testing | Blastula |
| N25-12-G | Blastula |  | Testing | Blastula |
| N25-1-G | Blastula |  | Testing | Blastula |
| N25-3-B | Nonblastula | Training |  |  |
| N25-10-B | Nonblastula | Training |  |  |
| N26-7-G | Blastula |  | Testing | Blastula |
| N26-3-G | Blastula |  | Testing | Blastula |
| N26-5-B | Nonblastula | Training |  |  |
| N26-4-B | Nonblastula | Training |  |  |
| N27-3-G | Blastula |  | Testing | Blastula |
| N27-2-G | Blastula |  | Testing | Blastula |
| N28-8-G | Blastula |  | Testing | Blastula |
| N29-13-G | Blastula |  | Testing | Nonblastula |
| N29-9-G | Blastula |  | Testing | Blastula |
| N29-8-G | Blastula |  | Testing | Blastula |
| N29-5-G | Blastula |  | Testing | Blastula |
| N29-3-B | Nonblastula | Training |  |  |
| N29-1-B | Nonblastula | Training |  |  |
| N31-9-B | Nonblastula | Training |  |  |
| N31-6-B | Nonblastula | Training |  |  |
| N32-8-G | Blastula |  | Testing | Blastula |
| N32-6-G | Blastula |  | Testing | Blastula |
| N32-5-G | Blastula |  | Testing | Blastula |
| N32-4-G | Blastula |  | Testing | Blastula |
| N32-2-G | Blastula | Training |  |  |
| N33-3-B | Nonblastula | Training |  |  |
| N33-2-B | Nonblastula | Training |  |  |
| N34-7-G | Blastula | Training |  |  |
| N34-1-B | Nonblastula | Training |  |  |
| N34-18-G | Blastula | Training |  |  |
| N34-16-G | Blastula | Training |  |  |
| N34-14-B | Nonblastula | Training |  |  |
| N34-10-G | Blastula | Training |  |  |
| N35-7-G | Blastula | Training |  |  |
| N35-6-G | Blastula | Training |  |  |
| N35-2-G | Blastula | Training |  |  |
| N35-1-G | Blastula | Training |  |  |
| N36-9-G | Blastula | Training |  |  |
| N36-7-G | Blastula | Training |  |  |
| N36-6-G | Blastula | Training |  |  |
| N36-4-G | Blastula | Training |  |  |
| N36-12-G | Blastula | Training |  |  |
| N37-7-G | Blastula | Training |  |  |
| N37-4-B | Nonblastula | Training |  |  |
| N37-1-B | Nonblastula | Training |  |  |
| N38-9-B | Nonblastula | Training |  |  |
| N38-6-G | Blastula | Training |  |  |
| N38-5-B | Nonblastula | Training |  |  |
| N38-15-G | Blastula | Training |  |  |
| N38-14-G | Blastula | Training |  |  |
| N38-13-G | Blastula | Training |  |  |
| N38-12-G | Blastula | Training |  |  |
| N39-4-B | Nonblastula | Training |  |  |
| N40-2-B | Nonblastula | Training |  |  |
| N40-1-B | Nonblastula | Training |  |  |
| N40-16-B | Nonblastula | Training |  |  |
| N41-4-G | Blastula | Training |  |  |
| N41-2-B | Nonblastula | Training |  |  |
| N42-8-G | Blastula | Training |  |  |

Supplementary Table 2. Clinical characteristics of patients in two groups.

|  | **Blastula** | **Non-blastula** | ***P*** |
| --- | --- | --- | --- |
| **Female** |  |  |  |
| Age | 35.30±3.77 | 40.29±3.35 | 0.013 |
| BMI (kg/m^2^) | 21.95±1.81 | 22.57±2.18 | 0.534 |
| Antral Follicle Count | 22.60±6.79 | 15.57±5.62 | 0.040 |
| AMH (ng/mL) | 5.01±3.63 | 5.27±2.94 | 0.879 |
| **Male** |  |  |  |
| Age | 41.40±8.37 | 43.29±5.19 | 0.606 |
| BMI (kg/m^2^) | 24.60±2.63 | 24.38±2.68 | 0.871 |
| Sperm Concentration (10^6^/ml) | 66.11±74.62 | 53.19±26.36 | 0.692 |
| Progressive Motility (%) | 39.12±17.16 | 31.93±23.22 | 0.488 |
| Normal Morphology (%) | 3.14±1.22 | 2.80±0.79 | 0.566 |
